# Performance of a prognostic 31-gene expression profile in an independent cohort of 523 cutaneous melanoma patients

**DOI:** 10.1186/s12885-018-4016-3

**Published:** 2018-02-05

**Authors:** Jonathan S. Zager, Brian R. Gastman, Sancy Leachman, Rene C. Gonzalez, Martin D. Fleming, Laura K. Ferris, Jonhan Ho, Alexander R. Miller, Robert W. Cook, Kyle R. Covington, Kristen Meldi-Plasseraud, Brooke Middlebrook, Lewis H. Kaminester, Anthony Greisinger, Sarah I. Estrada, David M. Pariser, Lee D. Cranmer, Jane L. Messina, John T. Vetto, Jeffrey D. Wayne, Keith A. Delman, David H. Lawson, Pedram Gerami

**Affiliations:** 1 Department of Cutaneous Oncology, Moffitt Cancer Center, 10920 N. McKinley Drive room 4123, Tampa, FL 33612 USA; 2 Department of Plastic Surgery, Cleveland Clinic Lerner Research Institute, 9500 Euclid Avenue, Cleveland, OH 44195 USA; 3 Department of Dermatology, Knight Cancer Institute, Oregon Health & Science University, 3303 S.W. Bond Avenue, Portland, OR 97239 USA; 4 Department of Medical Oncology, University of Colorado School of Medicine, 12801 E. 17th Avenue, Aurora, CO 80045 USA; 5 Department of Surgical Oncology, The University of Tennessee Health Science Center, 910 Madison, Suite 303, Memphis, TN 38163 USA; 6 Department of Dermatology, University of Pittsburgh Medical Center, 3601 Fifth Avenue, Pittsburgh, PA 15213 USA; 7 Department of Pathology, University of Pittsburgh Medical Center, 3708 Fifth Avenue, Suite 500.94, Pittsburgh, PA 15213 USA; 8 START Center for Cancer Care, 4383 Medical Drive, San Antonio, TX 78229 USA; 9 Castle Biosciences, Inc., 820 S. Friendswood Drive, Suite 201, Friendswood, TX 77546 USA; 10 Dermatology North Palm Beach, 840 U.S. Highway Number One, North Palm Beach, FL 33408 USA; 11 Research & Development, Kelsey Research Foundation, 5615 Kirby Drive, Suite 660, Houston, TX 77005 USA; 12 Affiliated Dermatology, 20401 North 73rd Street, Suite 230, Scottsdale, AZ 85255 USA; 13 Pariser Dermatology Specialists, Virginia Clinical Research, Inc., 6160 Kempsville Circle, Suite 200A, Norfolk, VA 23502 USA; 14 Eastern Virginia Medical School, P.O. Box 1980, Norfolk, VA 23501-1980 USA; 15 Department of Sarcoma Medical Oncology, Seattle Cancer Care Alliance, 825 Eastlake Avenue E, Seattle, WA 98109 USA; 16 Department of Anatomic Pathology, Moffitt Cancer Center, 10920 N. McKinley Drive, Tampa, FL 33612 USA; 17 Division of Surgical Oncology, Knight Cancer Institute, Oregon Health & Science University, 3303 S.W. Bond Avenue, Portland, OR 97239 USA; 18 Department of Surgical Oncology, Northwestern University Feinberg School of Medicine, 251 East Huron Street, Chicago, IL 60611 USA; 19 Department of Dermatology, Northwestern University Feinberg School of Medicine, 676 North St. Clair Street, Suite 1600, Chicago, IL 60611 USA; 20 Skin Cancer Institute, Northwestern University, Lurie Comprehensive Cancer Center, 420 East Superior Street, Chicago, IL 60611 USA; 21 Department of Surgery, Emory University Winship Cancer Institute, 1364 Clifton Road NE, Atlanta, GA 30322 USA; 22 Department of Hematology and Medical Oncology, Emory University Winship Cancer Institute, 550 Peachtree Street NE, Atlanta, GA 30308 USA; 23 Departments of Dermatology and Pathology, Northwestern University Feinberg School of Medicine, 676 North St. Clair Street, Arkes 1600, Chicago, IL 60611 USA

**Keywords:** Gene expression profiling, DecisionDx-Melanoma, Cutaneous melanoma, Metastasis, Prognosis, Staging

## Abstract

**Background:**

The heterogeneous behavior of patients with melanoma makes prognostication challenging. To address this, a gene expression profile (GEP) test to predict metastatic risk was previously developed. This study evaluates the GEP’s prognostic accuracy in an independent cohort of cutaneous melanoma patients.

**Methods:**

This multi-center study analyzed primary melanoma tumors from 523 patients, using the GEP to classify patients as Class 1 (low risk) and Class 2 (high risk). Molecular classification was correlated to clinical outcome and assessed along with AJCC v7 staging criteria. Primary endpoints were recurrence-free (RFS) and distant metastasis-free (DMFS) survival.

**Results:**

The 5-year RFS rates for Class 1 and Class 2 were 88% and 52%, respectively, and DMFS rates were 93% versus 60%, respectively (*P* < 0.001). The GEP was a significant predictor of RFS and DMFS in univariate analysis (hazard ratio [HR] = 5.4 and 6.6, respectively, *P* < 0.001 for each), along with Breslow thickness, ulceration, mitotic rate, and sentinel lymph node (SLN) status (*P* < 0.001 for each). GEP, tumor thickness and SLN status were significant predictors of RFS and DMFS in a multivariate model that also included ulceration and mitotic rate (RFS HR = 2.1, 1.2, and 2.5, respectively, *P* < 0.001 for each; and DMFS HR = 2.7, 1.3 and 3.0, respectively, *P* < 0.01 for each).

**Conclusions:**

The GEP test is an objective predictor of metastatic risk and provides additional independent prognostic information to traditional staging to help estimate an individual’s risk for recurrence. The assay identified 70% of stage I and II patients who ultimately developed distant metastasis. Its role in consideration of patients for adjuvant therapy should be examined prospectively.

**Electronic supplementary material:**

The online version of this article (10.1186/s12885-018-4016-3) contains supplementary material, which is available to authorized users.

## Background

Cutaneous melanoma continues to be a significant contributor to cancer morbidity and mortality, with over 90,000 new cases and over 9000 deaths expected in 2018 [1]. Assessment of survival outcomes is based on the American Joint Committee on Cancer (AJCC) staging [2]. Stage I and II patients greatly outnumber later stage patients, thus the vast majority of melanoma-related deaths occur in patients belonging to this group at diagnosis [3]. In the Multicenter Selective Lymphadenectomy Trial (MSLT-1), 13% of node-negative patients had biologically aggressive disease that resulted in metastases and death [3, 4]. The fact that a substantial proportion of melanoma related deaths occur in patients with thin, T1, melanoma tumors has also been reported [5–7]. Based on current guidelines these patients do not receive the intensive surveillance or adjuvant therapy offered to AJCC high risk patients [8]. Recent advances in our understanding of tumor biology should enable us to identify high-risk disease based on molecular characteristics of the tumor [9–11].

A 31-gene expression profile (GEP) test that dichotomizes cutaneous melanoma patients as Class 1 (low-risk) or Class 2 (high-risk) has been previously described [12, 13]. Class 2 results are associated with an increased risk for metastatic disease that is independent of staging factors [12]. This study evaluates the GEP test in a previously unreported, independent cohort of 523 cutaneous melanoma cases from a multi-center consortium.

## Methods

### Cohort selection

Following institutional review board approval of the study and waiver of patient consent at each of the 16 participating centers, archival formalin-fixed, paraffin-embedded primary cutaneous melanoma tumor tissue was collected. Inclusion in the study required biopsy confirmed stage I–III cutaneous melanoma diagnosed between 2000 and 2014, with at least 5 years of follow-up, unless there was an earlier documented recurrence or metastatic event. Thus, all cases diagnosed after October 31, 2011 that were included in the study had a documented metastatic event. All cases included in the study that had no documented metastasis event had at least 5 years of follow-up. Clinical, pathological and outcome data were collected by collaborating centers through an electronic case report form, and on-site monitoring of each case was completed prior to data analysis with a censor date of October 31, 2016.

### Data collection and class assignment

Expression profiling of the 31 genes (28 class-discriminating and 3 endogenous control genes; Additional file 1: Table S1) was performed via RT-PCR and radial basis machine (RBM) predictive modeling was used to generate a probability score and subsequent class assignment (Class 1 or Class 2) for each sample, as previously described [12, 13]. Only cases that met preestablished pre- and post-analytic quality control thresholds were included (Table 1).

**Table 1:** Clinical characteristics of the cohort

Clinical Characteristics	*n* = 523
Median age (range), years	59 (18–92)
Median follow-up for patients without a metastatic event, years (range)	7.5 (5.0–16.5)
Recurrence/distant metastasis	142/111
Median time to first recurrence, years (range)	1.2 (0.0–10.0)
AJCC stage	
I (total)	264 (50%)
IA	108
IB	76
Unknown substage^a^	80
II (total)	93 (18%)
IIA	35
IIB	26
IIC	17
Unknown substage	15
III (total)	166 (32%)
IIIA	69
IIIB	57
IIIC	35
Unknown substage	5
Breslow thickness	
Median (range), mm	1.2 (0.1–29.0)
≤ 1 mm	223 (43%)
> 1 mm	296 (56%)
Unreported	4 (1%)
Mitotic index	
< 1/mm^2^	99 (19%)
≥ 1/mm^2^	240 (46%)
Unreported	184 (35%)
Ulceration	
Absent	309 (59%)
Present	133 (26%)
Unreported	81 (15%)
SLN status	
Untested	186 (36%)
Negative	180 (34%)
Positive	157 (30%)
GEP Class	
Class 1	314 (60%)
Class 2	209 (40%)

*SLN* sentinel lymph node, *GEP* gene expression profile

^a^Substage information was not available in clinical documentation for these patients

The RBM model generates a linear probability score from 0 to 1. Within the model, cases with a probability score between 0 and 0.49 are labeled Class 1, with samples within one standard deviation (SD) of the median probability score for Class 1 cases (0–0.41) designated as Class 1A and samples outside of the SD (0.42–0.49) designated as Class 1B (Additional file 2: Supplemental methods). Similarly, Class 2 cases have a score between 0.5 and 1. Samples with a probability score within one SD of the median (0.59–1) are classified as Class 2B, while those with a score outside the SD (0.5–0.58) are labeled Class 2A. In both the Class 1 and Class 2 groups, “A” subclass reflects a better and “B” reflects a worse prognosis within the Class. Results from subclass analysis are reported in the clinical setting.

Primary endpoints were recurrence-free survival (RFS), or time from diagnosis to any local, regional, or distant recurrence, excluding a positive SLN, and distant metastasis-free survival (DMFS), or time from diagnosis to any distant metastasis. Melanoma-specific survival (MSS), or time from diagnosis to death documented as resulting from melanoma, was a secondary endpoint. All survival variables were calculated from documented diagnosis and event (or censor) dates.

### Statistical analysis

Kaplan-Meier and Cox proportional hazards survival analyses were performed using R version 3.3.0, with *P* < 0.05 considered statistically significant by log-rank method or Cox regression analysis. For proportional hazards analysis, Breslow thickness was measured as a continuous variable, while all other factors were dichotomized.

## Results

### Performance of the GEP

**Fig. 1:**
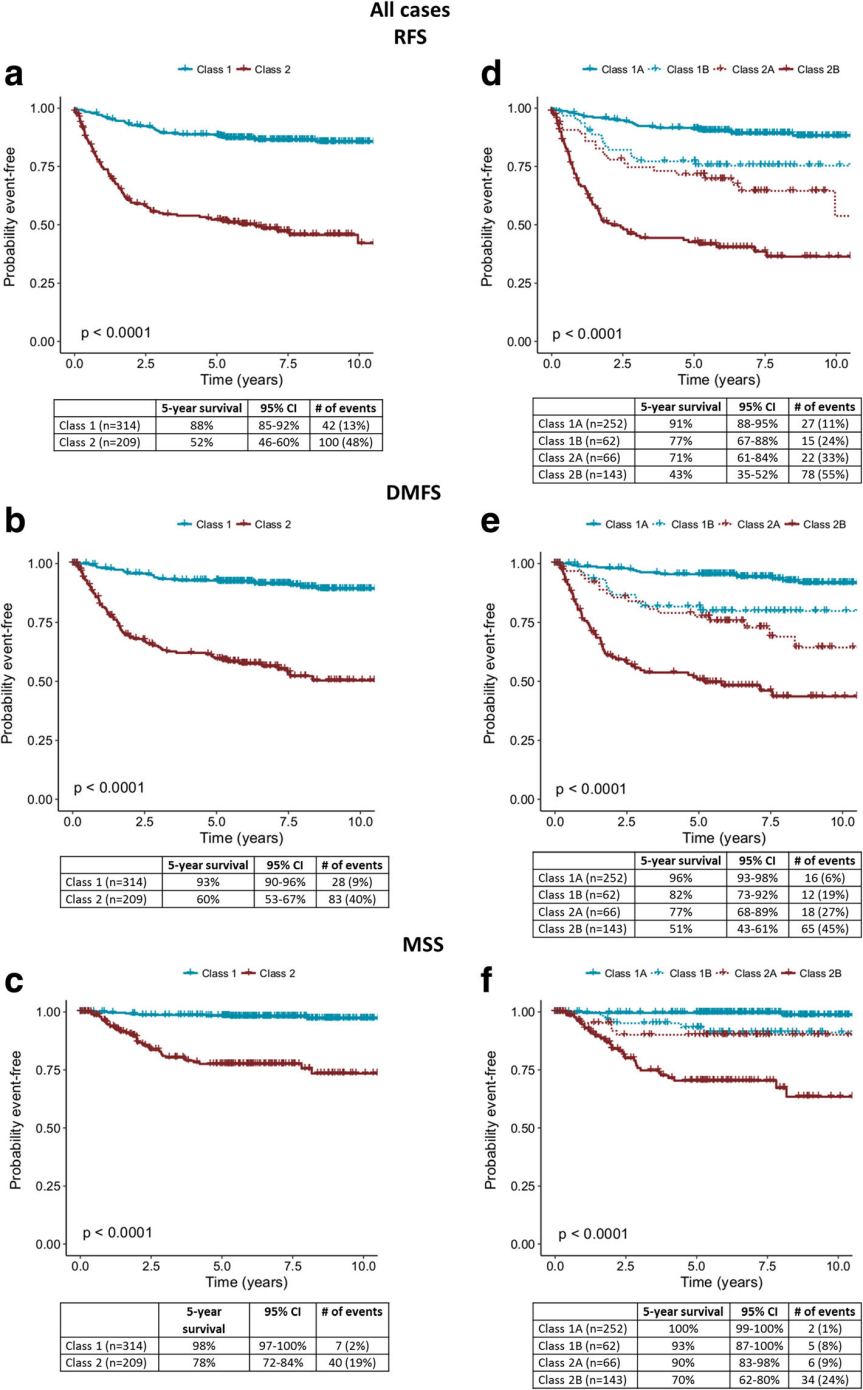
Gene expression profile class and correlated survival outcomes of the 523 patient cohort. **a** Recurrence-free, **b** distant metastasis-free, and **c** melanoma-specific survival rates for 523 patients using binary classification as indicated by Kaplan-Meier analysis. **d** Recurrence-free, **e** distant metastasis-free, and f melanoma-specific survival rates for 523 patients using molecular subclassification. Five-year survival rates, number of specified events, 95% confidence intervals, and percentages of each class experiencing an event are listed in the tables below the curves

Of 601 cutaneous melanoma cases, 523 met inclusion criteria (Table 1). Class 1 patients had 5-year RFS, DMFS and MSS rates of 88%, 93% and 98% in Kaplan-Meier analysis, respectively, compared to 52%, 60% and 78% for the Class 2 group (*P* < 0.001 for all comparisons; Fig. 1a-c). Analysis of survival rates by molecular substage resulted in Class 1A RFS, DMFS and MSS of 91%, 96% and 100%, respectively, compared to Class 2B rates of 43%, 51% and 70%, respectively (*P* < 0.001; Fig. 1d-f).

**Fig. 2:**
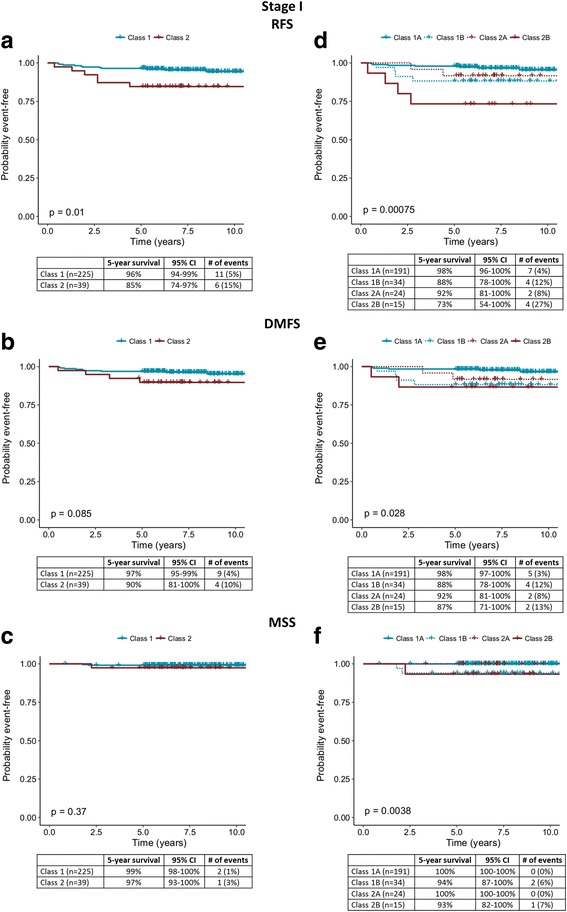
Survival outcomes for stage I patients with molecular classification by the 31-gene expression profile test. **a** Recurrence-free, **b** distant metastasis-free, and **c** melanoma-specific survival rates for stage I cases (*n* = 264) using binary classification as indicated by Kaplan-Meier analysis. **d** Recurrence-free, **e** distant metastasis-free, and **f** melanoma-specific survival rates for 264 stage I cases using molecular subclassification. Five-year survival rates, number of specified events, 95% confidence intervals, and percentages of each class experiencing an event are listed in the tables below the curves

Kaplan-Meier analysis for stage I showed 5-year RFS rates for all Class 1 and 2 patients of 96% and 85% (*P* = 0.01, Fig. 2a). By comparison, considering the risk associated with GEP subclasses, RFS rates of 98% and 73% were observed for Class 1A and Class 2B groups, respectively (*P* < 0.001 [adjusted], *P* = 0.0008 [nominal], Fig. 2d). DMFS rates for Class 1 and Class 2 groups were 97% and 90%, respectively (*P* = 0.085; Fig. 2b), while DMFS rates for Class 1A and Class 2B groups were 98% and 87%, respectively (*P* = 0.05 [adjusted], *P* = 0.028 [nominal], Fig. 2e). MSS rates for Class 1A and Class 2B groups were 100% and 93%, respectively (*P* < 0.01 [adjusted], *P* = 0.0038 [nominal], Fig. 2f).

**Fig. 3:**
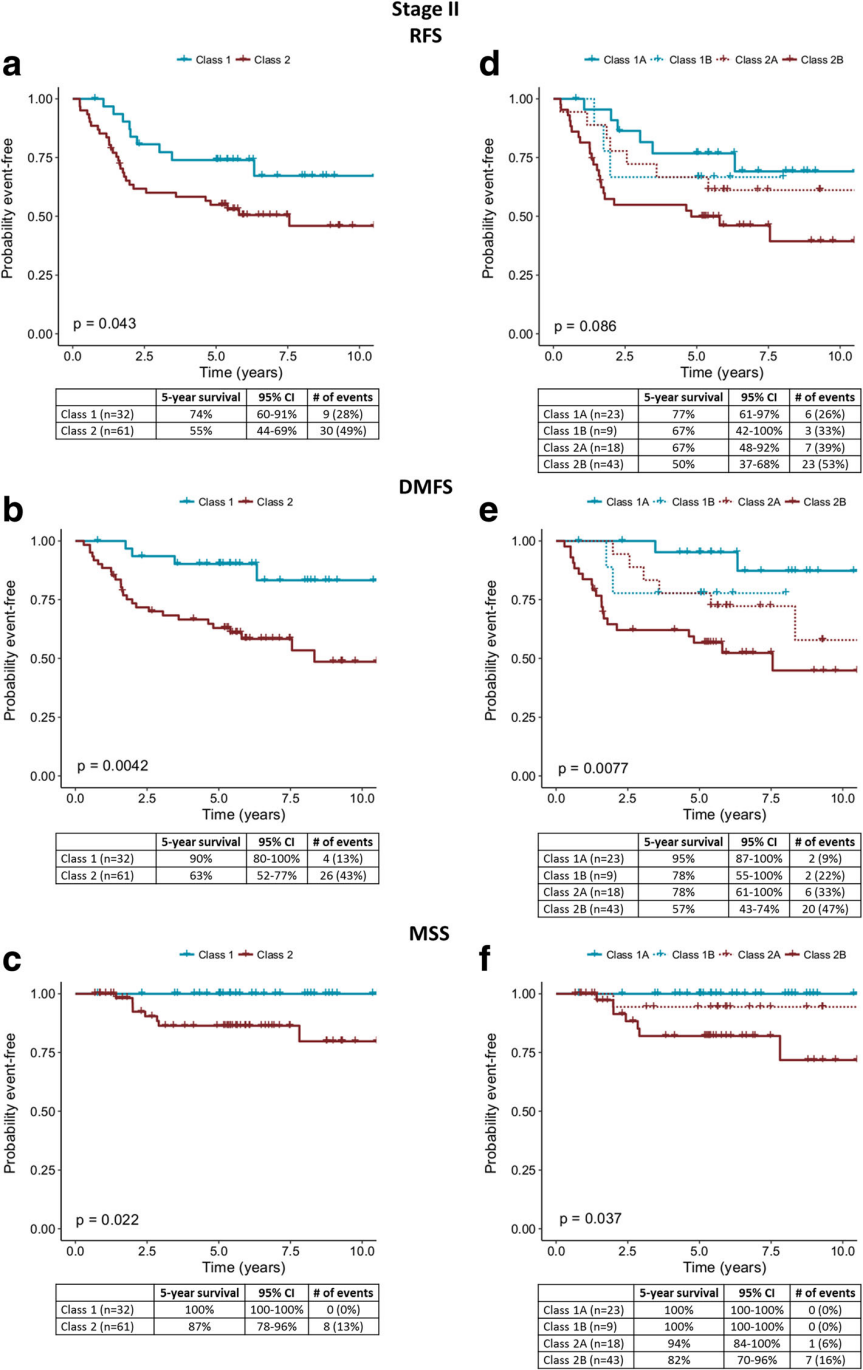
Survival outcomes for stage II patients with molecular classification by the 31-gene expression profile test. **a** Recurrence-free, **b** distant metastasis-free, and **c** melanoma-specific survival rates for stage II cases (*n* = 93) using binary classification as indicated by Kaplan-Meier analysis. **d** Recurrence-free, **e** distant metastasis-free, and **f** melanoma-specific survival rates for stage II cases using molecular subclassification. Five-year survival rates, number of specified events, 95% confidence intervals, and percentages of each class experiencing an event are listed in the tables below the curves

In stage II, 5-year RFS rates were 74% and 55% (*P* = 0.043, Fig. 3a), and DMFS rates were 90% and 63% (*P* = 0.004, Fig. 3b), respectively, for Class 1 and 2 patients. Comparing Class 1A and 2B groups, 5-year RFS rates were 77% and 50% (*P* = 0.13 [adjusted], *P* = 0.086 [nominal], Fig. 3d), and DMFS rates were 95% and 57%, respectively (*P* < 0.001 [adjusted], *P* = 0.0077 [nominal], Fig. 3e). MSS rates for Class 1A and Class 2B were 100% and 82%, respectively (*P* = 0.13 [adjusted], *P* = 0.037 [nominal], Fig. 2f). Of note, 30 of 43 stage I and II patients (70%) who had a distant metastasis were Class 2 (Table 2). Of the 11 stage I and II patients who died from melanoma, 9 (82%) were Class 2.

**Table 2:** Distant metastasis according to stage and molecular class in the stage I and II patients

Stage	Total cases	No Distant Metastasis			With Distant Metastasis		
		Total	Class 1	Class 2	Total	Class 1	Class 2
I^a^/IA/IB	264	251	216	35	13	9	4
II^a^	15	11	4	7	4	0	4
IIA	35	25	15	10	10	2	8
IIB	26	18	7	11	8	1	7
IIC	17	9	2	7	8	1	7
Total	357	314	244	70	43	13	30

^a^Substage unknown

There were 166 stage III cases in the study. Stage IIIA patients had 5-year RFS rates for Class 1 and 2 of 72% and 51%, respectively (*P* = 0.015, Additional file 3: Figure S1A), DMFS rates of 80% and 54% (*P* = 0.019, Additional file 3: Figure S1B), and MSS rates of 100% and 67% (*P* = 0.009, Additional file 3: Figure S1C).

### GEP independently predicts metastatic risk

In univariate Cox regression analysis, Breslow thickness, mitotic rate, ulceration, positive SLN, and molecular Class 2 were all significant predictors of recurrence and distant metastasis. In multivariate analysis, molecular Class 2, Breslow thickness, and positive SLN were independent predictors of RFS and DMFS (Table 3). The expanded confidence GEP subclasses were also significant predictors of RFS and DMFS in both multivariate and univariate models (Additional file 4: Table S2).

**Table 3:** Multivariate Cox regression analysis for recurrence and distant metastasis based on 244 cases with complete data for all variables

	Univariate			Multivariate^a^		
	HR	95% CI	*P* value	HR	95% CI	*P* value
RFS						
Breslow	1.3	1.2–1.3	< 0.001	1.2	1.1–1.3	< 0.001
Mitotic rate ≥ 1/mm2	3.3	1.9–5.7	< 0.001	0.9	0.5–1.7	0.8
Ulceration present	4.5	3.2–6.5	< 0.001	1.4	0.8–2.2	0.2
SLN positive	3.5	2.4–5.1	< 0.001	2.5	1.6–4.0	< 0.001
GEP Class 2	5.4	3.7–7.7	< 0.001	2.1	1.3–3.4	0.003
DMFS						
Breslow	1.4	1.3–1.5	< 0.001	1.3	1.2–1.4	< 0.001
Mitotic rate ≥ 1/mm2	3.9	2.0–7.5	< 0.001	0.9	0.5–2.0	0.9
Ulceration present	4.8	3.2–7.2	< 0.001	1.2	0.7–2.1	0.5
SLN positive	3.8	2.5–5.9	< 0.001	3.0	1.7–5.2	< 0.001
GEP Class 2	6.6	4.3–10.2	< 0.001	2.7	1.5–4.8	0.002

*CI* confidence interval, *DMFS* distant metastasis-free survival, *GEP* gene expression profile, *RFS* recurrence-free survival

^a^The multivariate Cox regression model includes data from 244 of 523 cases with complete information for Breslow thickness, mitotic rate, ulceration, SLN status and GEP class

### Evaluation with SLN biopsy status

Of the 523 cases evaluated, 337 had confirmed results from both the GEP test and SLN biopsy (SLNB). In comparing SLN-negative/Class 1 patients with SLN-negative/Class 2 patients, the 5-year RFS was 87% vs. 67%, DMFS was 93% vs. 75%, and MSS was 98% vs. 92% (Table 4). For SLN-positive/Class 1, the RFS, DMFS and MSS rates were 61%, 74% and 93%, respectively, while in SLN-positive/Class 2 patients’ rates were 37%, 44% and 63%, respectively. The expanded GEP subclasses were also significant in association with SLN status (Additional file 5: Table S3). SLN-negative/Class 1A vs. SLN-negative/Class 2B cases had 90% vs. 60%, 96% vs. 69%, and 100% vs. 88% 5-year RFS, DMFS, and MSS rates, respectively. SLN-positive/Class 1A vs. SLN-positive/Class 2B cases had 60% vs. 32%, 76% vs 38%, and 97% vs.59% 5-year RFS, DMFS, and MSS rates respectively.

**Table 4:** Recurrence-free, distant metastasis-free, and melanoma-specific survival rates in the population of patients receiving a sentinel lymph node biopsy

	RFS (# events, 95% CI)	DMFS (# events, 95% CI)	MSS (# events, 95% CI)
Class 1 (*n* = 159)	79% (37, 72–85%)	87% (24, 82–93%)	97% (7, 94–100%)
Class 2 (*n* = 178)	51% (89, 44–59%)	59% (74, 51–67%)	78% (35, 71–85%)
SLN- (*n* = 180)	79% (43, 73–85%)	85% (32, 80–91%)	95% (9, 92–99%)
SLN+ (*n* = 157)	47% (82, 39–56%)	55% (66, 47–65%)	75% (33, 68–84%)
Class 1/SLN- (*n* = 103)	87% (15, 81–94%)	93% (9, 88–98%)	98% (2, 95–100%)
Class 1/SLN+ (*n* = 56)	61% (22, 49–76%)	74% (15, 63–88%)	93% (5, 86–100%)
Class 2/SLN- (*n* = 77)	67% (28, 57–79%)	75% (23, 66–85%)	92% (7, 85–98%)
Class 2/SLN+ (*n* = 101)	37% (60, 28–49%)	44% (51, 34–56%)	63% (28, 52–76%)

*CI* confidence interval, *DMFS* distant metastasis-free survival, *GEP* gene expression profile, *RFS* recurrence-free survival, *SLN* sentinel lymph node, MSS melanoma-specific survival

### Accuracy of the GEP compared to SLN biopsy

**Fig. 4:**
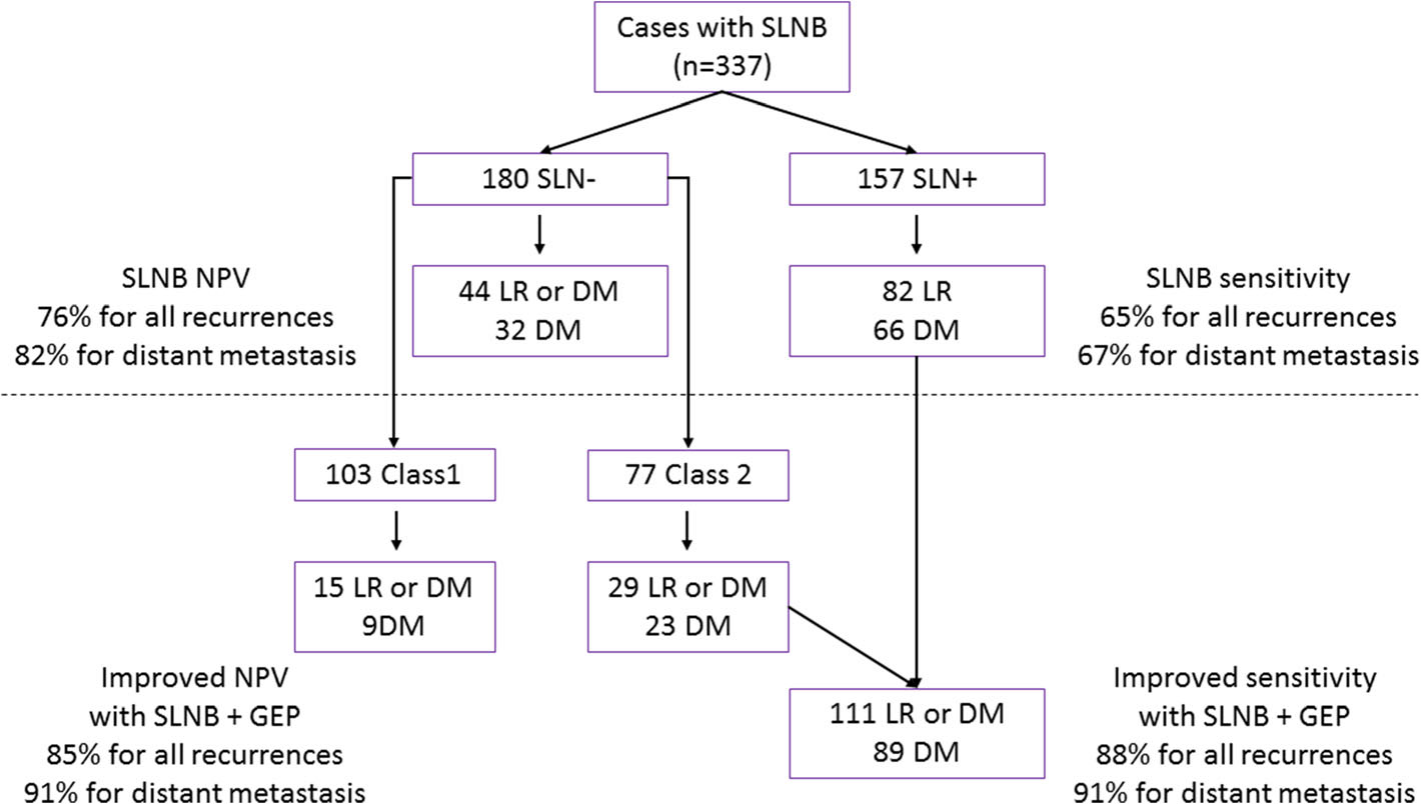
Clinical utility of gene expression profiling with sentinel lymph node biopsy (SLNB). A schematic of the enhanced identification of high-risk melanoma patients when gene expression profiling is used in combination with SLNB prognostication. With SLNB only, sensitivities for all recurrences [local recurrence (LR) and distant metastasis (DM)] or distant metastases only (DM) are 65% or 67%, respectively (above dotted line). Inclusion of GEP identifies as high risk an additional 29 recurrences and 23 distant metastases, improving overall sensitivity of recurrences to 88%, and sensitivity of distant metastases to 91%. Similarly, the negative predictive value (NPV) is also improved when combining SLNB with the GEP test

Class 2 results showed sensitivity of 70% for prediction of recurrence, 75% for distant metastasis, and 85% for melanoma-specific death, compared to the sensitivity of SLN-positivity of 66%, 67% and 79%, respectively (Table 5). A schematic depicting the clinical utility of the GEP is presented in Fig. 4, showing improved sensitivity for prediction of both locoregional (LR) and distant metastasis (DM) when the test is used in combination with SLNB. The specificity of a Class 1 result for recurrence, distant metastasis, and melanoma-specific death were 71%, 69%, and 64% compared to 65%, 62%, and 58% for SLN negativity. The positive predictive values (PPV) of a Class 2 signature and SLN-positivity, were 48% and 52% for recurrence, 40% and 42% for distant metastasis, and 19% and 21% for melanoma-specific mortality. The PPV of a Class 2B was 55% for recurrence, 45% for distant metastasis, and 24% for melanoma-specific mortality (Additional file 6: Table S4). The negative predictive values (NPV) of the Class 1 signature and a SLN-negative result were 87% and 76% for recurrence, 91% and 82% for distant metastasis, and 98% and 95% for melanoma-specific mortality. The NPV of a Class 1A was 89% for recurrence, 94% for distant metastasis and 99% for melanoma-specific mortality (Additional file 6: Table S4).

**Table 5:** Accuracy of the GEP test and sentinel lymph node status

	GEP Class	SLN status
	% (95% CI)	% (95% CI)
RFS		
Sensitivity	70% (62–78%)	66% (57–74%)
Specificity	71% (67–76%)	65% (58–71%)
PPV	48% (41–55%)	52% (44–60%)
NPV	87% (82–90%)	76% (69–82%)
DMFS		
Sensitivity	75% (66–83%)	67% (57–76%)
Specificity	69% (65–74%)	62% (55–68%)
PPV	40% (33–47%)	42% (34–50%)
NPV	91% (87–94%)	82% (76–88%)
MSS		
Sensitivity	85% (72–94%)	79% (63–90%)
Specificity	64% (60–69%)	58% (52–64%)
PPV	19% (14–25%)	21% (15–28%)
NPV	98% (95–99%)	95% (91–98%)

*CI* confidence interval, *DMFS* distant metastasis-free survival, *GEP* gene expression profile, *MSS* melanoma-specific survival, *NPV* negative predictive value, *PPV* positive predictive value, *RFS* recurrence-free survival, *SLN* sentinel lymph node

## Discussion

The use of molecular classification of disease is now routine in clinical practice [10, 14]. For any new molecular clinical test it is critical to evaluate whether the test i) accurately predicts its intended outcome; ii) has consistent, sustainable accuracy across multiple independent studies, and iii) adds value beyond existing clinical tools [15–17]. Here we report that the 31-gene expression profile test is able to predict metastatic risk in an independent cohort of 523 melanoma patients with results that are consistent with those reported in prior studies [12, 13]. In this cohort, we observed a 5-year DMFS rate of 93% for Class 1 cases and 62% for Class 2 cases (compared to 100% and 58%, respectively, in the smaller, initial study). We previously reported that this test could identify the majority of SLN-negative patients with an elevated risk of metastasis [12]. In this study, the majority (70%) of the node-negative patients who had a distant metastasis were Class 2, as well as the majority (78%) of SLN-negative patients who died from melanoma (7 of 9 patients).

This study is based on a cohort of melanoma patients with clinical characteristics that align with those of the general cutaneous melanoma population. While the SLN positivity rate is higher than the 15–20% reported in previous studies, the 5-year survival rates for the SLN-negative and SLN-positive groups (95% vs. 75%, respectively) are similar to those reported in the MSLT-1 study (90% vs. 70%, respectively) [3, 4]. Breslow thickness, ulceration and mitotic rate were all important in univariate models of risk prediction (Table 3), supporting similarity with previous cohorts used to identify relevant staging factors. SLN status is currently regarded as the gold standard for prognosticating cutaneous melanoma, as a positive SLNB is associated with a significantly increased risk of metastasis [4] and our results confirm this. Compared to the SLNB procedure, the GEP test performed with better sensitivity across all endpoints studied. The results suggest that the GEP could enhance current prognostic accuracy by identifying clinically and pathologically SLN-negative patients who harbor an elevated risk of metastasis. Thus, highest sensitivity for detecting patients at high risk for recurrence, distant metastasis or melanoma-specific death can be achieved when the test is used in combination with current staging criteria. Importantly, this is coupled with high negative predictive values across endpoints, reflecting a substantially low risk associated with the Class 1 result. While the positive predictive values are lower, this accuracy metric may be impacted by 1) a favorable host immune response to metastatic tumor cells; and 2) follow-up time that is not long enough to observe the metastatic event. Importantly, the positive predictive values observed for the GEP are similar to those observed SLN status in this cohort (Table 5).

Considering that approximately two thirds of melanoma-related deaths in patients originally diagnosed without distant metastatic disease (stage I-III) occur in SLN negative patients (stage I-II) [3], the identification of patients in this group with biologically aggressive disease is a clinically significant unmet need. The current study demonstrates that implementing the GEP test after initial staging of melanoma tumors adds value by further stratifying the risk associated with stage I and stage II patients. That value is illustrated by a risk of recurrence that is three times higher for the stage I/Class 2 group compared to the stage I/Class 1 group (15% vs. 5%), and nearly seven times higher when comparing the stage I/Class 2B group to the stage I/Class 1A group (27% vs. 4%). The stage II/Class 2 group has nearly twice the risk of recurrence compared to the stage II/Class 1 group (49% vs. 28%), however, it should be noted that five of the nine events in the Class 1 group were regional recurrences. By comparison, the stage II/Class 2 group has three times the risk of developing distant metastasis compared to the stage II/Class 1 group (43% vs. 13%) and five times the risk in the stage II/Class 2B group compared to the stage II/Class 1A group (47% vs. 9%). The ability to subdivide stage II patients into groups with as high as 43% chance of developing distant metastasis and alternatively groups with as low as 5% risk at 5-years could significantly impact management decisions and clinical care. The results suggest that the GEP offers the opportunity to personalize risk assessment within each of these population-based AJCC stages.

The identification of high risk early stage patients is especially relevant considering current advances in melanoma therapies, which require us to improve risk evaluation in order to better weigh benefit versus harm of adjuvant therapy [18]. These findings suggest that new tools are necessary to supplement current staging approaches, even as we achieve better outcomes for melanoma patients overall. Early stage patients could potentially benefit from adjuvant therapy but may not be recognized as high risk by the current staging system, and even among stage III patients there is often a dilemma as to whether systemic treatment is appropriate. The results of this study suggest that the GEP test should be evaluated in the context of new adjuvant therapy trials and trials evaluating the benefit of management approaches in stage III patients.

One of the limitations of this study is the inclusion of samples in the cohort that were diagnosed prior to widespread standardization of reporting for pathological variables such as Breslow thickness, ulceration and mitosis and therefore some pathology reports did not specify all features. However, the Cox regression models assessing the association between GEP and those factors account for this limitation and only patients with all factors specified were included in this analysis. Another limitation is the retrospective nature of the study and thus does not take into account recent advances in management of patients with advanced melanoma in the adjuvant and metastatic settings. However, recently published results of an interim analysis of the GEP test in a prospective cohort show consistency of results with this another retrospective cohorts [12, 13, 19].

Current guidelines indicate that management should ultimately be tailored to an individual’s probability of recurrence [20]. The risk classification provided by this test, along with current prognostic factors, can be used to better estimate an individual’s risk for recurrence and therefore aid in determining the most appropriate surveillance methodology and frequency. As illustrated in Fig. 4, the clinical utility of the test in conjunction with SLNB can identify as many as 89% of the patients who will experience a distant metastasis, and over 70% of those patients who are SLNB-negative. Several recent studies have demonstrated that modern therapies for melanoma are more effective when disease burden is low [21, 22]. Thus, the need to accurately predict risk in melanoma patients is more critical than ever to enable risk-tailored surveillance and management of early staged patients with biologically aggressive tumors.

## Conclusions

The 31-gene expression profile is an accurate predictor of metastatic risk that has shown consistent performance and provides additional prognostic information to standard clinical and pathologic factors included in AJCC staging.

## Additional files

Additional file 1: Table S1. Control and discriminant gene targets assessed by the GEP test. (DOCX 12 kb)

Additional file 2: Supplemental Methods Supplemental Methods for the 523-patient cohort. These methods describe the generation of four subclasses of GEP test results (1A, 1B, 2A, 2B) based on the linear probability score. (PDF 260 kb)

Additional file 3: Figure S1. Survival outcomes for stage IIIA and combined stage IIIB & IIIC patients with molecular classification by the 31-gene expression profile test. (PDF 343 kb)

Additional file 4: Table S2. Cox regression analysis for recurrence and distant metastasis incorporating reduced confidence groups. The multivariate model is based on 244 cases with complete data for all variables assessed. (DOCX 13 kb)

Additional file 5: Table S3 Survival rates combining normal and reduced confidence GEP results with SLN status in the population of patients receiving a sentinel lymph node biopsy. (DOCX 12 kb)

Additional file 6: Table S4. Accuracy of the GEP test, limiting GEP result to the normal confidence Class 1A or Class 2B groups. (DOCX 13 kb)

## Abbreviations

AJCC: American Joint Committee on Cancer
CI: Confidence interval
DM: Distant metastasis
DMFS: Distant metastasis-free survival
GEP: Gene expression profile
LR: Locoregional recurrence
MSLT-1: Multicenter Selective Lymphadenectomy Trial
MSS: Melanoma-specific survival
NPV: Negative predictive value
PPV: Positive predictive value
RBM: Radial basis machine
RFS: Recurrence-free survival
SD: Standard deviation
SLN: Sentinel lymph node
SLNB: Sentinel lymph node biopsy
