# Supplementary material for: Performance of a prognostic 31-gene expression profile in an independent cohort of 523 cutaneous melanoma patients
Source: BMC Cancer. 2018 Feb 5;18:130. doi: 10.1186/s12885-018-4016-3 (PMC5800282; doi:10.1186/s12885-018-4016-3)
Supplement: Supplementary file 1 — Control and discriminant gene targets assessed by the GEP test. (DOCX 12 kb) [file 12885_2018_4016_MOESM1_ESM.docx]

**eTable 1.** Control and discriminant gene targets assessed by the GEP test.

| **Control genes** |  |  |
| --- | --- | --- |
| HNRPNL | YKT6 | FXR1 |
| **Discriminant genes** |  |  |
| BAP1* | MGP | SPP1 |
| CXCL14 | CLCA2 | S100A8 |
| BTG1 | SAP130 | ARG1 |
| KRT6B | GJA1 | ID2 |
| EIF1B | S100A9 | CRABP2 |
| KRT14 | ROBO1 | RBM23 |
| TACSDT2 | DSC1 | SPRR1B |
| TRIM29 | AQP3 | TYRP1 |
| PPL | LTA4H | CST6 |

*Two assays for BAP1 are included to target both the 5’ and 3’ regions of the gene
