# Supplementary material for: Performance of a prognostic 31-gene expression profile in an independent cohort of 523 cutaneous melanoma patients
Source: BMC Cancer. 2018 Feb 5;18:130. doi: 10.1186/s12885-018-4016-3 (PMC5800282; doi:10.1186/s12885-018-4016-3)
Supplement: Supplementary file 2 — Supplemental Methods for the 523-patient cohort. These methods describe the generation of four subclasses of GEP test results (1A, 1B, 2A, 2B) based on the linear probability score. (PDF 260 kb) [file 12885_2018_4016_MOESM2_ESM.pdf]

Supplemental Methods for the 523-patient cohort

Class normal and reduced confidence molecular subclassifications

The radial basis machine model generates a linear probability score of 0-1, with 0.5 serving as the cutoff between Class 1 and Class 2 (eFigure 3). An area of reduced biological resolution around the cutoff was defined at the time of establishment of the initial training set[1]. One standard deviation (SD) from the median probability score of Class 1 patients without evidence of metastasis in the training set, and the median probability score of the Class 2 patients with evidence of metastasis in the training set, established the cutoff points for a "normal" confidence limit (Class 1A and Class 2B, respectively). Values outside of these SDs were considered with "reduced" confidence in class assignment (Class 1B and Class 2A). Class 1A, 1B, 2A and 2B results are reported in the clinical setting.

Study endpoints for the cumulative cohort

The primary endpoints evaluated for this study were: i) recurrence-free survival (RFS), or the time from diagnosis to any local, regional, or distal recurrence, excluding a positive SLN; and ii) distant metastasis-free survival (DMFS), or the time from diagnosis to any distant metastasis; and iii) melanoma-specific survival (MSS), or the time from diagnosis to death from melanoma, was also analyzed.

References

1. Lawson DH CR, Johnson C, Russell MC, Amaria RN, Wilkinson J, Gerami P, et al. Continued evaluation of a 31-gene expression profile test (GEP) for prediction of distant metastasis (DM) in cutaneous melanoma (CM). J Clin Oncol. 2015;33 Suppl 15:9066.
