# Supplementary material for: Performance of a prognostic 31-gene expression profile in an independent cohort of 523 cutaneous melanoma patients
Source: BMC Cancer. 2018 Feb 5;18:130. doi: 10.1186/s12885-018-4016-3 (PMC5800282; doi:10.1186/s12885-018-4016-3)
Supplement: Supplementary file 3 — Survival outcomes for stage IIIA and combined stage IIIB & IIIC patients with molecular classification by the 31-gene expression profile test. (PDF 343 kb) [file 12885_2018_4016_MOESM3_ESM.pdf]

eFigure 1. Survival outcomes for stage IIIA and stage IIIB and C patients with molecular classification by the 31-gene expression profile test. A) Recurrence-free survival, B) distant metastasis-free survival, and C) melanoma-specific survival for Class 1 and 2 subjects with stage IIIA disease (n=69) from the 523-sample cohort. D) Recurrence-free survival, E) distant metastasis-free survival, and F) melanoma-specific survival for Class 1 and 2 subjects with stage IIIB and IIIC disease (n=92) from the 523-sample cohort. Tables below graphs provide five-year survival rates with 95% confidence intervals and number of events. Class 1 shown in blue, Class 2 shown in red.

Stage IIIA

A

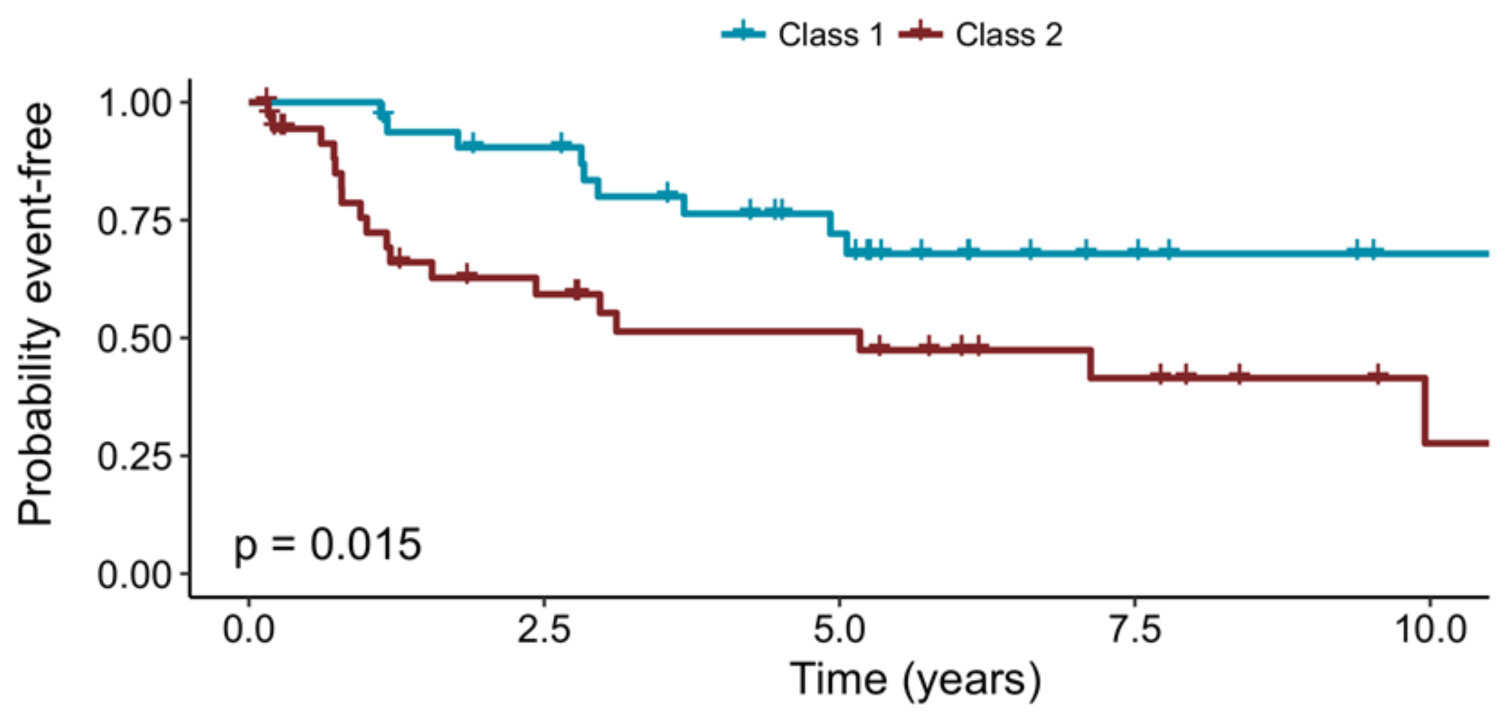

	5-year survival	95% CI	# of events
Class 1 (n=32)	72%	57-91%	9 (28%)
Class 2 (n=37)	51%	36-73%	18 (49%)

B

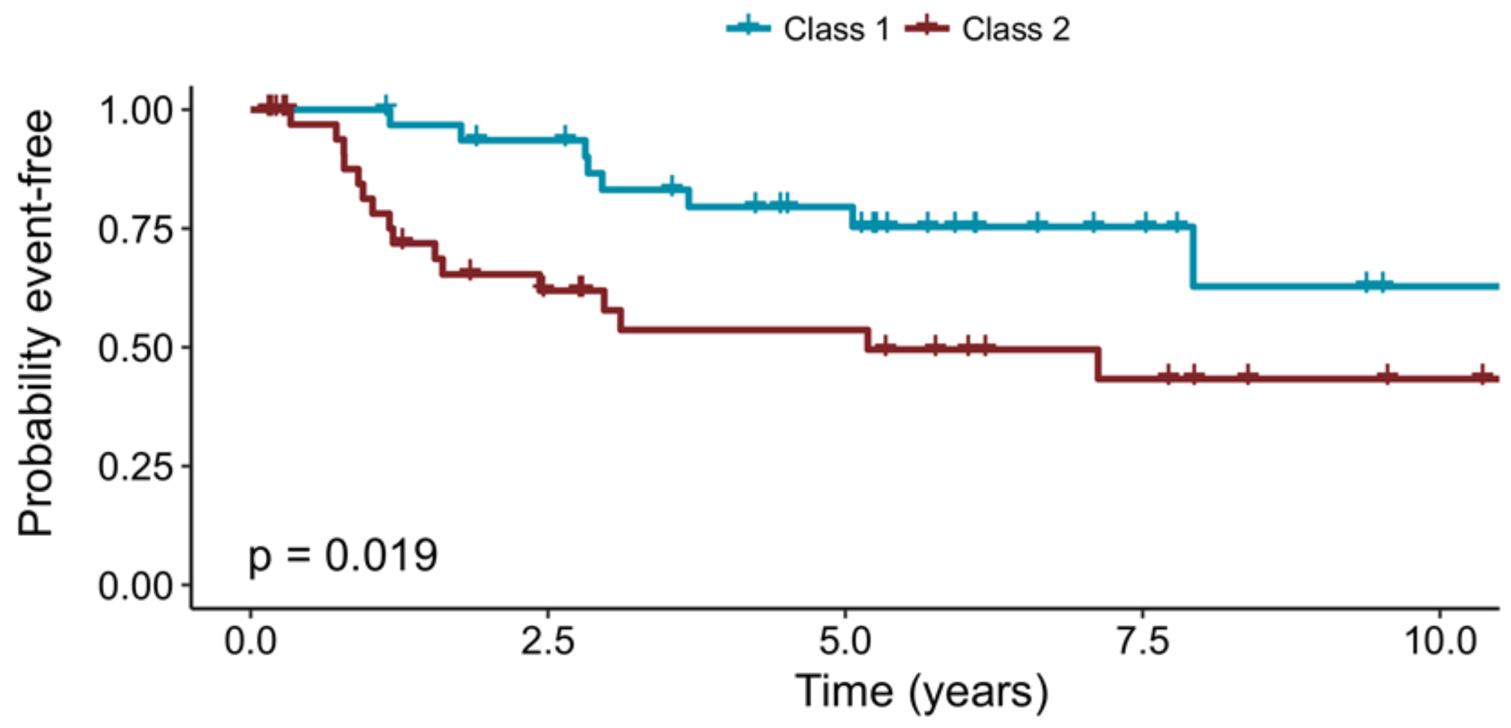

	5-year survival	95% CI	# of events
Class 1 (n=32)	80%	66-96%	8 (19%)
Class 2 (n=37)	54%	38-75%	16 (38%)

C

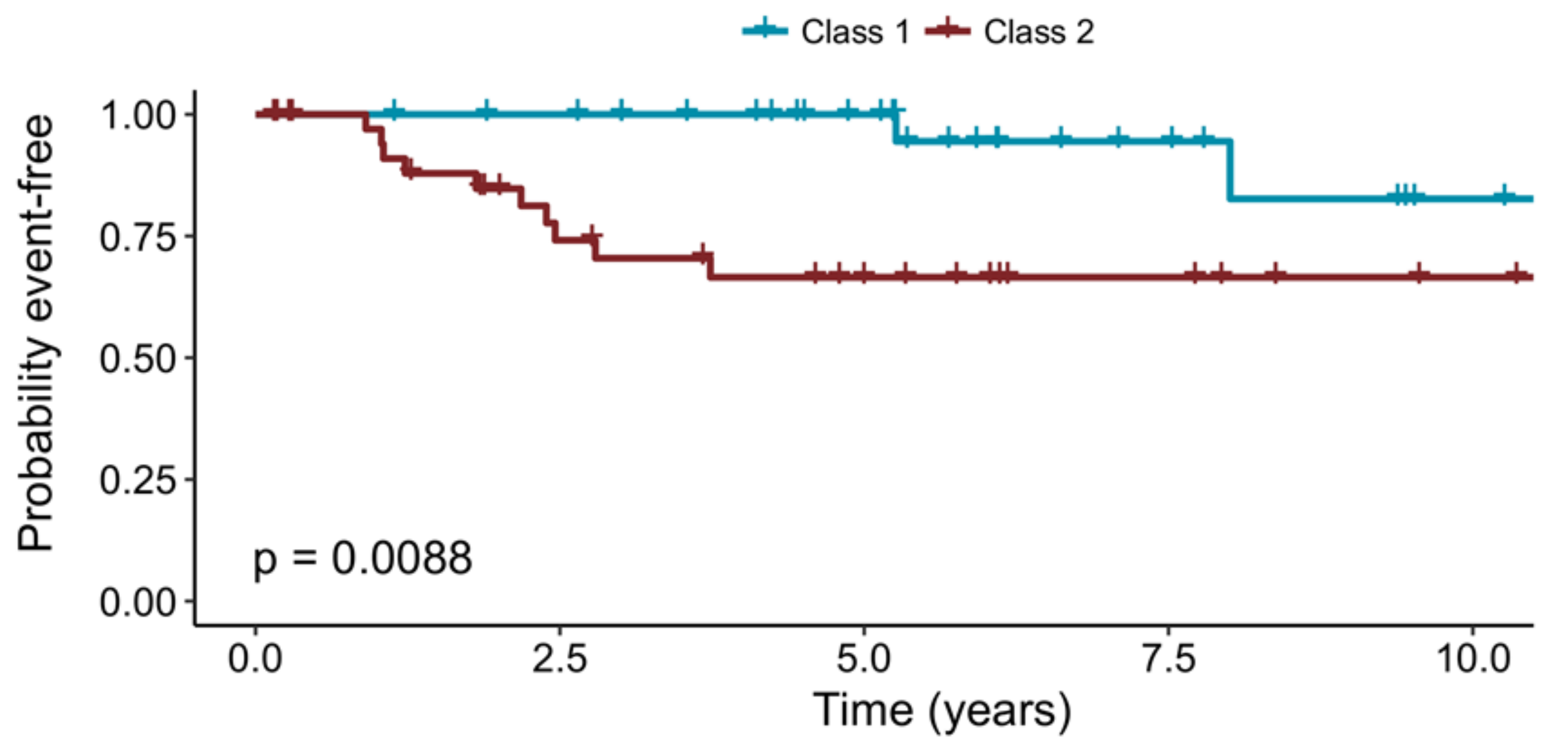

	5-year survival	95% CI	# of events
Class 1 (n=32)	100%	100-100%	2 (6%)
Class 2 (n=37)	67%	51-86%	10 (27%)

Stage IIIB and C

D

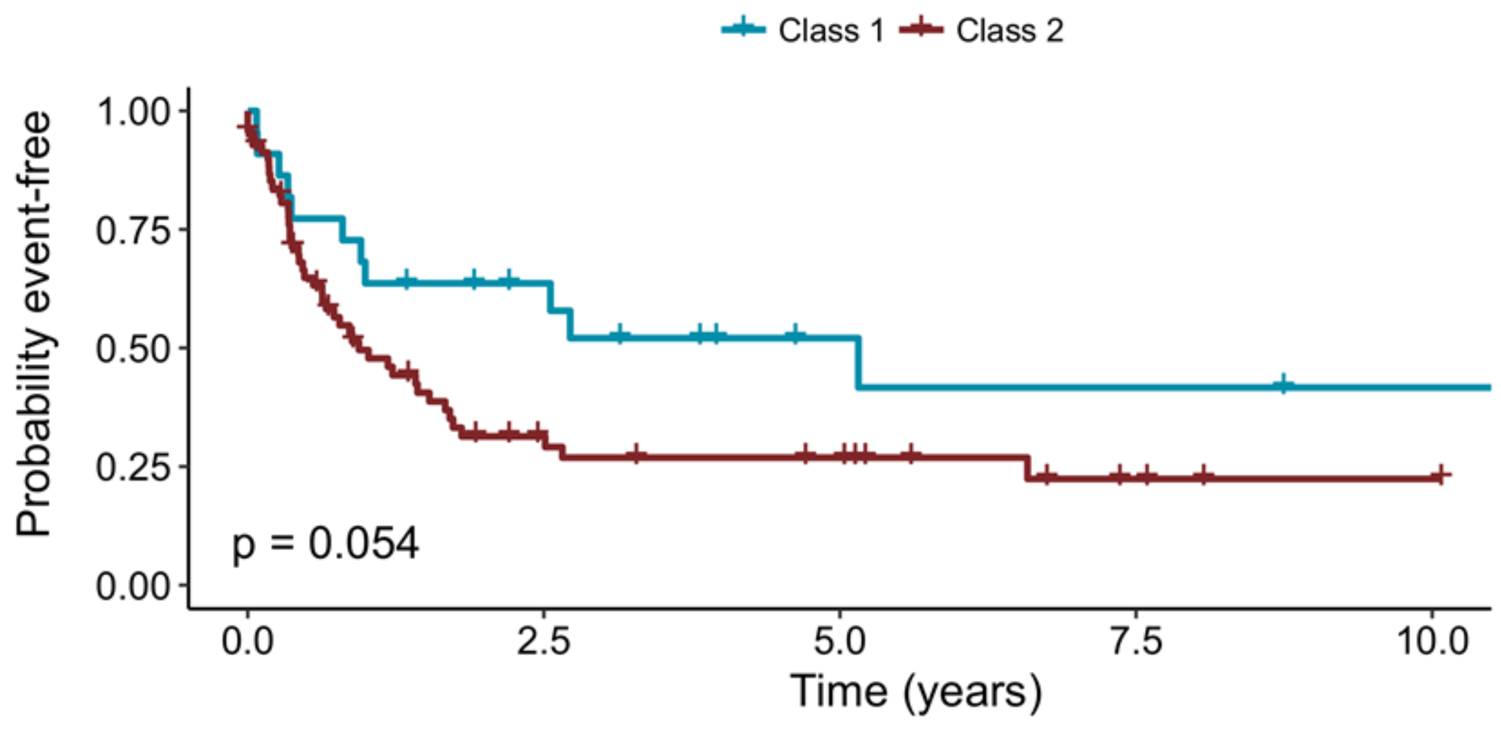

	5-year survival	95% CI	# of events
Class 1 (n=22)	52%	34-79%	11 (50%)
Class 2 (n=70)	27%	17-42%	45 (64%)

E

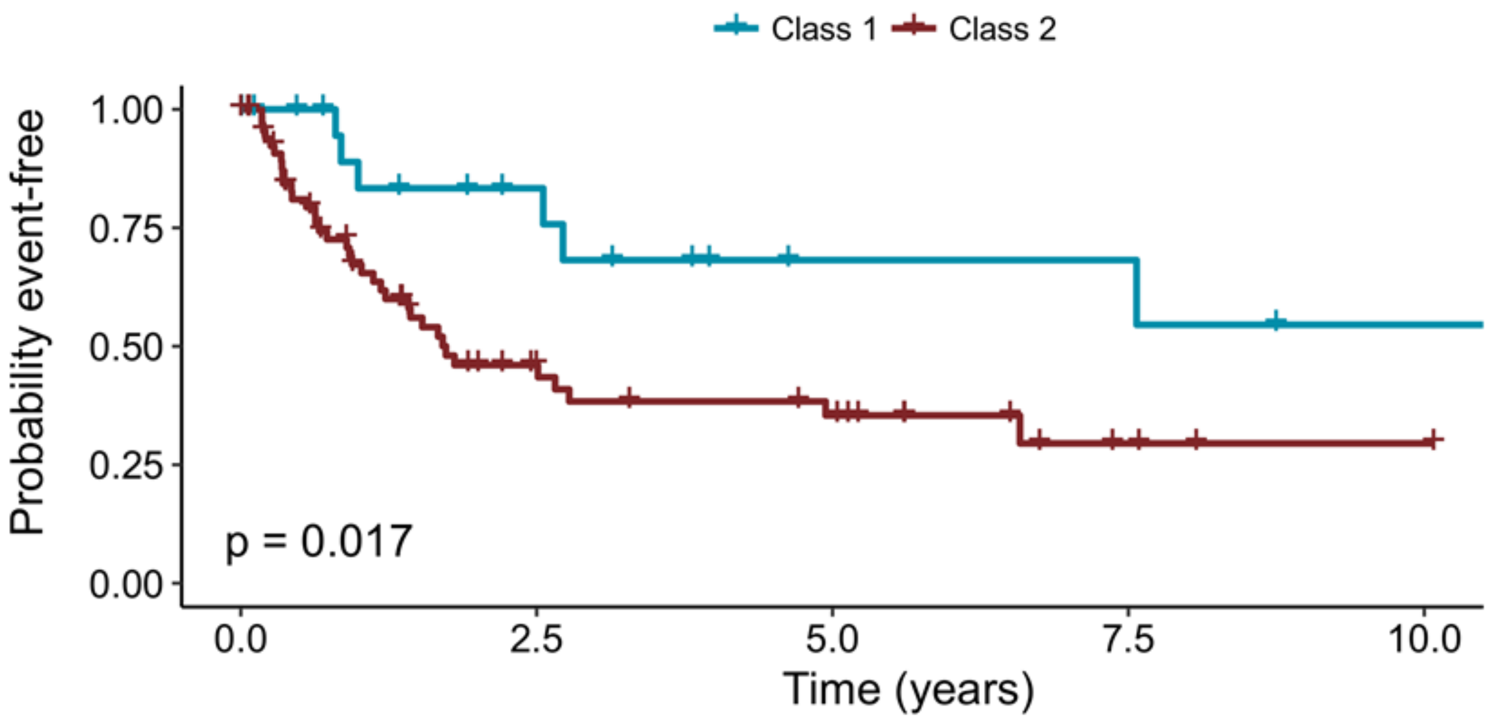

	5-year survival	95% CI	# of events
Class 1 (n=22)	68%	48-96%	6 (27%)
Class 2 (n=70)	35%	24-52%	36 (51%)

F

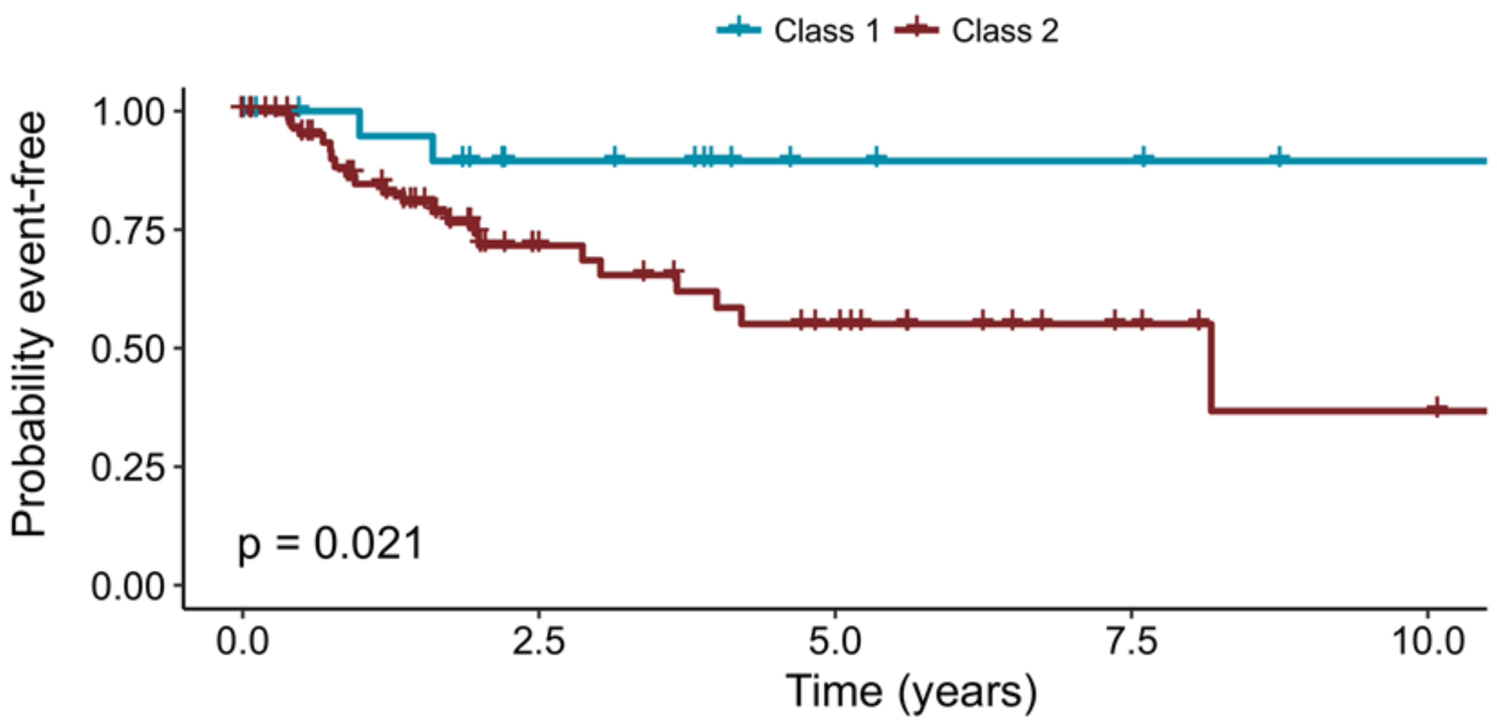

	5-year survival	95% CI	# of events
Class 1 (n=22)	89%	77-100%	2 (9%)
Class 2 (n=70)	55%	41-74%	21 (30%)
