# Supplementary material for: Performance of a prognostic 31-gene expression profile in an independent cohort of 523 cutaneous melanoma patients
Source: BMC Cancer. 2018 Feb 5;18:130. doi: 10.1186/s12885-018-4016-3 (PMC5800282; doi:10.1186/s12885-018-4016-3)
Supplement: Supplementary file 4 — Cox regression analysis for recurrence and distant metastasis incorporating reduced confidence groups. The multivariate model is based on 244 cases with complete data for all variables assessed. (DOCX 13 kb) [file 12885_2018_4016_MOESM4_ESM.docx]

**eTable 2.** Cox regression analysis for recurrence and distant metastasis in the 523-patient cohort, incorporating both reduced confidence Class 1B/2A and normal confidence Class 2B groups

|  | **Univariate** | | | **Multivariate*** | | |
| --- | --- | --- | --- | --- | --- | --- |
| **RFS** | **HR** | **95% CI** | ***P* value** | **HR** | **95% CI** | ***P* value** |
| Breslow | 1.3 | 1.2-1.3 | <0.001 | 1.2 | 1.1-1.3 | <0.001 |
| Mitotic rate ≥1/mm^2^ | 3.3 | 1.9-5.7 | <0.001 | 1.5 | 0.8-2.7 | 0.23 |
| Ulceration present | 4.5 | 3.2-6.5 | <0.001 | 1.3 | 0.8-2.0 | 0.27 |
| SLN positive | 3.5 | 2.4-5.1 | <0.001 | 2.6 | 1.7-4.0 | <0.001 |
| GEP Class 1B | 3.4 | 1.6-7.1 | <0.01 | 2.9 | 0.9-4.1 | 0.0818 |
| GEP Class 2A | 5.1 | 2.6-10.0 | <0.001 | 2.1 | 1.0-4.5 | 0.0485 |
| GEP Class 2B | 12.7 | 7.3-22.0 | <0.001 | 3.8 | 2.0-6.9 | <0.001 |
| **DMFS** | **HR** | **95% CI** | ***P* value** | **HR** | **95% CI** | ***P* value** |
| Breslow | 1.4 | 1.3-1.5 | <0.001 | 1.3 | 1.2-1.4 | <0.001 |
| Mitotic rate ≥1/mm^2^ | 3.9 | 2.0-7.5 | <0.001 | 1.3 | 0.6-2.7 | 0.5 |
| Ulceration present | 4.8 | 3.2-7.2 | <0.001 | 1.1 | 0.7-1.9 | 0.7 |
| SLN positive | 3.8 | 2.5-5.9 | <0.001 | 2.9 | 1.7-4.7 | <0.001 |
| GEP Class 1B | 10.6 | 2.1-54.6 | <0.01 | 2.6 | 1.0-6.7 | 0.0441 |
| GEP Class 2A | 13.5 | 2.7-67.1 | <0.01 | 2.9 | 1.1-7.4 | 0.0274 |
| GEP Class 2B | 47 | 11.3-196 | <0.001 | 5.3 | 2.5-11.6 | <0.001 |

*Multivariate Cox regression model includes Breslow, mitotic rate, ulceration, SLN status and GEP class

CI, confidence interval; DMFS, distant metastasis-free survival; GEP, gene expression profile; RFS, recurrence-free survival; SLN, sentinel lymph node
