# Supplementary material for: Performance of a prognostic 31-gene expression profile in an independent cohort of 523 cutaneous melanoma patients
Source: BMC Cancer. 2018 Feb 5;18:130. doi: 10.1186/s12885-018-4016-3 (PMC5800282; doi:10.1186/s12885-018-4016-3)
Supplement: Supplementary file 5 — Survival rates combining normal and reduced confidence GEP results with SLN status in the population of patients receiving a sentinel lymph node biopsy. (DOCX 12 kb) [file 12885_2018_4016_MOESM5_ESM.docx]

**eTable 3.** Survival rates combining normal and reduced confidence GEP results with SLN status in the population of patients receiving a sentinel lymph node biopsy from the 523-patient cohort

|  | **RFS (95% CI)** | **DMFS (95% CI)** | **MSS (95% CI)** |
| --- | --- | --- | --- |
| Class 1A/SLN- | 90% (83-97%) | 96% (92-100%) | 100% (100-100%) |
| Class 1B/SLN- | 79% (64-97%) | 83% (70-100%) | 91% (81-100%) |
| Class 2A/SLN- | 77% (64-94%) | 84% (72-98%) | 97% (91-100%) |
| Class 2B/SLN- | 60% (47-76%) | 69% (56-84%) | 88% (78-98%) |
| Class 1A/SLN+ | 60% (45-80%) | 76% (62-93%) | 97% (92-100%) |
| Class 1B/SLN+ | 62% (44-89%) | 71% (53-96%) | 87% (72-100) |
| Class 2A/SLN+ | 56% (38-82%) | 60% (42-86%) | 73% (55-97%) |
| Class 2B/SLN+ | 32% (22-46%) | 38% (27-53%) | 59% (47-75%) |

CI, confidence interval; DMFS, distant metastasis-free survival; GEP, gene expression profile; MSS, melanoma-specific survival; RFS, recurrence-free survival; SLN, sentinel lymph node
