# Supplementary material for: Performance of a prognostic 31-gene expression profile in an independent cohort of 523 cutaneous melanoma patients
Source: BMC Cancer. 2018 Feb 5;18:130. doi: 10.1186/s12885-018-4016-3 (PMC5800282; doi:10.1186/s12885-018-4016-3)
Supplement: Supplementary file 6 — Accuracy of the GEP test, limiting GEP result to the normal confidence Class 1A or Class 2B groups. (DOCX 13 kb) [file 12885_2018_4016_MOESM6_ESM.docx]

**eTable 4.** Accuracy of the GEP test in the 523-patient cohort, limiting GEP result to the normal confidence Class 1A or Class 2B groups.

|  | **% (95% CI)** |
| --- | --- |
| **RFS** | |
| PPV_Class 2B_ | 55% (46-63%) |
| NPV_Class 1A_ | 89% (85-93%) |
| **DMFS** | |
| PPV_Class 2B_ | 45% (37-54%) |
| NPV_Class 1A_ | 94% (90-96%) |
| **MSS** | |
| PPV_Class 2B_ | 24% (17-32%) |
| NPV_Class 1A_ | 99% (97-100%) |

CI, confidence interval; DMFS, distant metastasis-free survival; GEP, gene expression profile; MSS, melanoma-specific survival; NPV: negative predictive value; PPV: positive predictive value; RFS, recurrence-free survival
